# Perturbation-Based Balance Training Using Repeated Trips on a Walkway vs. Belt Accelerations on a Treadmill: A Cross-Over Randomised Controlled Trial in Community-Dwelling Older Adults

**DOI:** 10.3389/fspor.2021.702320

**Published:** 2021-08-20

**Authors:** Patrick Y. H. Song, Daina L. Sturnieks, Michael K. Davis, Stephen R. Lord, Yoshiro Okubo

**Affiliations:** ^1^Falls, Balance and Injury Research Centre, Neuroscience Research Australia, Sydney, NSW, Australia; ^2^Faculty of Medicine and Health, University of New South Wales, Sydney, NSW, Australia; ^3^College of Health Sciences, University of Delaware, Newark, DE, United States

**Keywords:** perturbation, balance training, older adults, gait, exercise, accidental fall

## Abstract

**Background:** Walkway and treadmill induced trips have contrasting advantages, for instance walkway trips have high-ecological validity whereas belt accelerations on a treadmill have high-clinical feasibility for perturbation-based balance training (PBT). This study aimed to (i) compare adaptations to repeated overground trips with repeated treadmill belt accelerations in older adults and (ii) determine if adaptations to repeated treadmill belt accelerations can transfer to an actual trip on the walkway.

**Method:** Thirty-eight healthy community-dwelling older adults underwent one session each of walkway and treadmill PBT in a randomised crossover design on a single day. For both conditions, 11 trips were induced to either leg in pseudo-random locations interspersed with 20 normal walking trials. Dynamic balance (e.g., margin of stability) and gait (e.g., step length) parameters from 3D motion capture were used to examine adaptations in the walkway and treadmill PBT and transfer of adaptation from treadmill PBT to a walkway trip.

**Results:** No changes were observed in normal (no-trip) gait parameters in both training conditions, except for a small (0.9 cm) increase in minimum toe elevation during walkway walks (*P* < 0.01). An increase in the margin of stability and recovery step length was observed during walkway PBT (*P* < 0.05). During treadmill PBT, an increased MoS, step length and decreased trunk sway range were observed (*P* < 0.05). These adaptations to treadmill PBT did not transfer to a walkway trip.

**Conclusions:** This study demonstrated that older adults could learn to improve dynamic stability by repeated exposure to walkway trips as well as treadmill belt accelerations. However, the adaptations to treadmill belt accelerations did not transfer to an actual trip. To enhance the utility of treadmill PBT for overground trip recovery performance, further development of treadmill PBT protocols is recommended to improve ecological authenticity.

## Introduction

Falls in older people are a major health issue associated with significant morbidity, mortality (James et al., 2020), and economic burden (Davis et al., 2010). One-third of community-dwelling older adults fall annually (Lord et al., 1993), of which, 10–20% will require hospitalisation for complications such as hip fracture (Rubenstein, 2006). Evidence for fall prevention interventions consistently shows combinations of balance and functional exercises reduce the rate of falls, with an average effect of 34% (Sherrington et al., 2019). However, it has been suggested that the effects of conventional balance exercise are limited due to a lack of “task-specificity” to the balance recovery responses required to prevent falls (Grabiner et al., 2014). This has led to the development of perturbation-based balance training (PBT) which is a task-specific intervention exposing participants to repeated unexpected perturbations to improve reactive balance control (Mansfield et al., 2015; Gerards et al., 2017). A recent clinical trial found that PBT using an instrumented treadmill incorporated into conventional physiotherapy significantly reduced injurious falls in daily life, compared to physiotherapy alone (Lurie et al., 2020). Furthermore, systematic reviews and meta-analyses of randomised controlled trials (RCTs) have shown that PBT reduces the rate of falls by ~50% in older adults and individuals with neurological conditions (Mansfield et al., 2015; Okubo et al., 2017).

Whilst the reported efficacy of PBT is promising, several important questions are yet to be answered. Many heterogeneous perturbation methods have been used to simulate and train reactive balance and the most effective method is unknown (Gerards et al., 2017; Okubo et al., 2017). Therapist-applied perturbations such as push, pull, and lean-and-release during stance have been used in clinical settings as they require minimal space and can be administered easily (Gerards et al., 2017; Mansfield et al., 2018). In contrast, overground perturbation systems with hidden tripping obstacles and low-friction surfaces have been generally used only in laboratory studies. These overground systems can more closely resemble “real-life” perturbations including trips and slips during gait (Pai et al., 2014; Okubo et al., 2019b; Wang et al., 2020), thus having the advantage of “task-specificity.” However, since many of these systems require a long walkway and overhead harness track, their clinical feasibility is limited.

In contrast, an instrumented treadmill can deliver sudden perturbations during gait through belt acceleration and therefore offer a viable method for administering clinically feasible trip- and slip-like PBT. A study in 166 community-dwelling older adults reported significant transfer of training effects from treadmill-based slip training to improvement in balance recovery responses following an overground slip (Wang et al., 2019). Since trips are the most common cause of falls in community-dwelling older adults (Berg et al., 1997), previous studies used several treadmill methods to evoke trip-like balance responses such as belt accelerations (McCrum et al., 2018), ankle cable pulls (break-and-release) (Epro et al., 2018), and dropping an obstacle onto the belt (King et al., 2019). Although treadmill belt accelerations do not involve obstruction of the swinging foot, they simulate the overall forward trunk rotation and stepping during a trip to a certain degree (Sessoms et al., 2014). Thus, belt accelerations have been used as part of PBT in recent studies (McCrum et al., 2018, 2020; Lurie et al., 2020; Gerards et al., 2021). Because treadmill accelerations do not require additional perturbation devices other than an instrumented treadmill, the clinical feasibility of this approach may be high. However, it is important to clarify whether PBT using treadmill belt accelerations can provide meaningful adaptation to balance recovery from an actual trip. To our knowledge, no previous studies have examined whether adaptations to PBT with treadmill belt accelerations can transfer to actual overground trips.

The aims of this study were to (i) compare the training adaptations to repeated overground trips and treadmill belt accelerations in community-dwelling older adults and (ii) determine if any adaptations gained during treadmill PBT transferred to improved responses to a naïve overground trip. Based on previous studies (Bhatt and Pai, 2009; Wang et al., 2012; Okubo et al., 2019b), we hypothesised that both PBT regimes would induce significant and similar adaptations in dynamic stability during a trip and that participants with prior treadmill PBT would have significantly better responses in the overground trip, compared to those without prior PBT.

## Methodology

### Study Design

This study was a randomised crossover trial comparing treadmill and overground PBT, conducted at Neuroscience Research Australia (19 July 2019–3 March 2020). The study protocol was approved by the University of New South Wales Human Research Ethics Committee (HC16227).

### Participants

Prospective participants were recruited via a research volunteer database. Eligibility criteria were aged 65+ years, living independently, ability to walk 20 min unassisted, and no neurological impairments or osteoporosis. Written informed consent was obtained from all the participants.

### Randomisation

**Figure 1:**
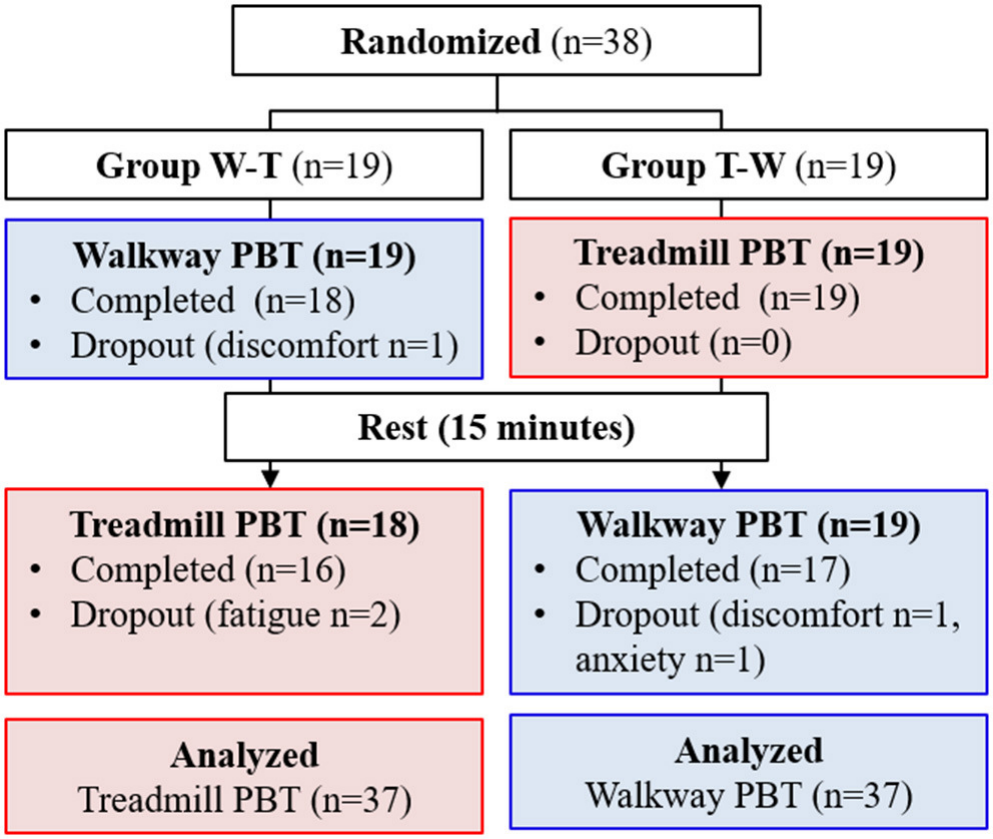
Flow chart of the cross-over randomised controlled trial.

Thirty-eight participants were randomly allocated into either Group W-T (*n* = 19) or Group T-W (*n* = 19) based on the flip of a coin. Group W-T completed the walkway PBT first followed by a 15-min break and then the treadmill PBT. In contrast, Group T-W completed the treadmill PBT before the break, followed by walkway PBT (Figure 1).

### Baseline Measurements

Participants were assessed regarding their concern about falling [Falls Efficacy Scale – International (Yardley et al., 2005)], mental health [Hospital Anxiety and Depression Scale (Snaith, 2003)], and falls in the past year.

### Experimental Protocol

Participants initially walked at the usual pace for three repeated trials over an 8-m course with a 5.7-m long electronic mat (GAITRite, CIR Systems, New Jersey, USA) to determine their step length, cadence, and gait speed to be used in the PBT conditions. In preparation for both walkway and treadmill PBT, participants were fitted with a ceiling-mounted full-body harness adjusted such that when hanging in the harness, their knees were 10 cm above the floor to prevent contact with the ground in the event of a fall.

### Walkway PBT Setup

Walkway PBT involved 11 trips and 18 normal walks on a custom-built 10 m wooden walkway (Supplementary Figure 1) (Okubo et al., 2018, 2019b). Target stepping tiles were placed along the walkway at 95% of individual usual step length, whilst a metronome was set to 95% of their usual cadence. During a 3-min practice and throughout training, participants were instructed to walk while stepping on the target tiles in time with the metronome beat, yielding a walking speed of 90% of their usual speed. If the gait of the participant did not match the metronome timing and stepping tile locations (by visual inspection), then additional familiarisation trials were undertaken.

Trips were induced by a 14 cm height spring-loaded tripping board which flipped up when activated by the participant moving over an optical foot detection sensor hidden in the walkway. The tripping board was positioned at the late-swing phase (at ~60–70% of the gait cycle from foot contact) to increase the likelihood of a lowering strategy (Eng et al., 1994) to induce a similar response to the treadmill condition (an elevating strategy never occurs on a treadmill). To minimise prediction of a trip, 18 normal walk (no-trip) trials were interspersed with 11 trip trials, presented in various locations (left or right side and near, middle, or far position) in a pre-determined, pseudo-random order (Table 1; Supplementary Figure 1). Participants were instructed that they may experience a hazard anywhere and at any time whilst walking on the walkway but to try to continue walking normally. To evaluate potential training effects in a consistent manner, the first (T1), fourth (T4), seventh (T7), and eleventh (T11) trips were delivered to the left leg in the middle of the walkway.

**Table 1:** The training protocol used for both treadmill and overground training.

**Trial type**	**Trip location**	**Tripped foot**	**Anxiety and perceived difficulty**
N1			Check
N2			
N3			
N4			
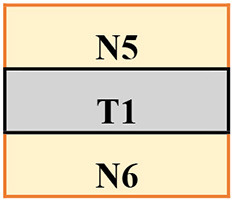			
			Check

	Middle	Left	
T2	Near	Left	
N7			
T3	Middle	Right	
N8			
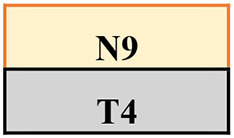			

	Middle	Left	
N10			
T5	Far	Left	Check
N11			
T6	Middle	Right	
N12			
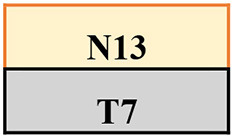			

	Middle	Left	
N14			
T8	Far	Left	Check
N15			
T9	Middle	Right	
N16			
T10	Near	Left	
N17			
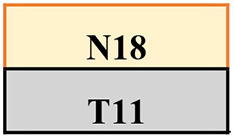			

	Middle	Left	Check

*“T” denotes a trip trial. “N” denotes a normal (no-trip) walk trial. Trials on the treadmill were conducted continuously, thus a series of 30 steps was considered a trial, equivalent to one return walk over the 10 m overground walkway. The trip location denotes the position of the tripping board on the walkway, which was not relevant on the treadmill because participants walk in place over the moving treadmill belt. Shaded trials were used for statistical analysis*.

### Treadmill PBT Setup

The treadmill PBT was conducted on a dual-belt, instrumented treadmill (M-Gait, Motekforce Link, Amsterdam, The Netherlands) controlled by custom-written software within D-Flow 3.30.2 (Motek Medical B.V., Amsterdam, The Netherlands) interfaced with an 8-camera Vicon motion capture system (Bonita, Vicon Motion Systems Ltd., Oxford, UK). During a 3-min practice period and throughout training, participants walked on the treadmill with the belt speed set to 90% of their individual walking speed. A perturbation was induced by a sudden acceleration of one side of the treadmill belt at 8 m/s^2^ to up to 200% of the walking belt speed. The belt acceleration began at approximately mid-swing of the gait cycle (triggered by a hallux marker of the to-be-perturbed limb passed the hallux marker of the stance limb in the sagittal plane) so that perturbation was delivered at the subsequent foot strike (McCrum et al., 2018). Each treadmill belt perturbation was delivered for 30% of stride taken from the average time of the previous three strides. Participants were instructed that they may experience a hazard at any time whilst walking but to try to continue walking normally. Similar to the walkway, treadmill PBT involved 11 belt accelerations interspersed with 18 (30–90 s) long bouts of normal (no-trip) walking. Each walk was however in a continuous sequence (Table 1). To minimise prediction, belt accelerations were induced to both left and right legs in a pre-determined pseudo-random order. The first (T1), fourth (T4), seventh (T7), and eleventh (T11) belt accelerations were induced on the left leg to be used for analysis.

### Outcome Measures

#### Falls Incidence and Recovery Strategy

A fall was defined by a post-trip harness supported load of >30% of the body weight of the participant (Yang and Pai, 2011) as measured by a load cell in series with the harness line. Walkway trip recovery strategies were classified as either a lowering strategy (i.e., when the obstructed foot immediately stepped down in front of the obstacle) or an elevating strategy (i.e., when the obstructed foot was elevated to clear the obstacle).

#### Kinematics

Eight-camera motion capture systems (Vicon Motion Systems Ltd., Oxford, UK) were used to collect 3D kinematic data during the treadmill (Bonita cameras) and walkway (Vantage cameras) PBT sessions. Thirty-nine 14-mm diameter retroreflective markers were attached to anatomical landmarks according to the Plug-in-Gait full-body model marker set (Vicon Motion Systems, 2017). Kinematic variables were calculated from sagittal-plane marker trajectories using custom software in MATLAB R2019b (The MathWorks, Inc., MA, USA) (see Supplementary Table 1 for detail).

To assess predictive and reactive gait adaptations during trip trials, the following kinematic parameters were calculated one step before (Pre1) and the first (Rec1), second (Rec2), and third (Rec3) steps after trip-onset (i.e., one previous and three recovery steps). On the walkway, the step that cleared the tripping board was treated as Rec1, that is, the tripped (left) footstep in an elevating strategy and the contralateral (right) footstep in the lowering strategy.

As a measure of dynamic stability, the margin of stability (MoS) in the anterior–posterior direction was calculated at step touchdown. The MoS is the distance (cm) between the closest edge (usually the toe) of the base of support and extrapolated centre of mass (*XCoM*), which accounts for the velocity of the *CoM* (Hof et al., 2005; Süptitz et al., 2013):

$$\begin{array}{l} XCoM\;=P_{CoM}+\;\frac{V_{CoM}+{\bar{V}}_{BoS}}{\sqrt{\frac{g}{L}}} \end{array}$$

where *P*_*CoM*_ is the position of the *CoM* estimated by the Dynamic Plug-in-Gait model (relative to the ankle marker of the trailing limb), *VCoM* is the velocity of the *CoM*, ${\bar{V}}_{BoS}$ is the velocity of the heel marker on the belt (averaged during stance phase), *g* is gravitational acceleration (9.81 m/s^2^), and *L* is the sagittal distance between the *CoM* and the ankle joint centre. *V*_*BoS*_ was assumed zero for walkway trials. A positive MoS indicates the *XCoM* is within the base of support and therefore a stable body configuration. A negative MoS indicates an unstable body configuration and a requirement to take additional steps to avoid a fall.

To quantify the magnitude of the balance perturbation, anteroposterior distance (cm) between *XCoM* and the rear ankle joint centre (marker) of the trailing limb was calculated at step touchdown. A positive value indicates the forward location of the *XCoM* relative to the rear foot ankle joint centre. Maximum toe elevation was also measured at previous and recovery steps. Trunk sway range was defined as maximal angular displacement of the trunk over one previous step or three recovery steps.

During normal walking trials, spatiotemporal gait parameters including step length, cadence, gait speed, and minimum toe elevation were calculated for subsequent analysis.

#### Self-Reported Anxiety and Difficulty Levels

Participants were asked to report their level of anxiety and perceived difficulty prior to N1, following N5 (prior to T1), T5, T8, and T11. Participants reported anxiety using a 5-point scale with one representing “not at all” and five representing “extremely anxious.” Participants reported their perceived difficulty in the last trial using a five-point scale with one representing “easy” and five representing “too hard.” The anxiety of the participants and perceived difficulty scores at the five time points were averaged for the analyses.

### Statistical Analysis

Approximate normality of variable distributions was confirmed with the Shapiro-Wilk test and visual inspection of Q–Q plots, and logarithmic (base 10) transformation for skewed data was conducted if required to allow parametric analysis. Changes in anxiety and perceived difficulty (N1 vs. N5/T5/T8/T11) during treadmill and walkway PBT were tested using Wilcoxon signed rank test with Bonferroni adjustments. Average anxiety and perceived difficulty scores for the trip trials (N1, N5, T5, T8, and T11) were also compared between the walkway vs. treadmill PBT using a paired *t*-test. Potential predictive gait adaptations during normal walks (N5 vs. N6/N9/N13/N18) were examined using the spatiotemporal gait parameters with a generalised linear mixed model with robust estimation (robust against violations of model assumptions) and sequential Bonferroni adjustments. Potential training effects (predictive and reactive gait adaptation) on pre- and post-trip kinematics were examined using a generalised linear mixed model with time (T1, T4, T7, and T11), step (Pre, Rec1, Rec2, and Rec3) and condition (treadmill, walkway) entered as factors and interaction effects adjusted for the group (i.e., training order). Changes from T1 to T4/T7/T11 within each step were examined by *post-hoc* pairwise comparisons with sequential Bonferroni corrections. Transfer of any training effects from treadmill PBT to a walkway trip was examined by comparing the first walkway trip (T1) parameters between Group W-T (prior to training) and Group T-W (after treadmill PBT) using independent-samples *t*-tests. All statistical tests were conducted using IBM SPSS Statistics 25 (IBM Corp., New York, USA). *P* < 0.05 was considered statistically significant.

## Results

### Participant Characteristics

Thirty-eight participants were recruited and randomised into Group W-T (*n* = 19) or Group T-W (*n* = 19). Five participants (13%) could not complete all the protocols due to fatigue (*n* = 2), discomfort (*n* = 1), and anxiety (*n* = 2). Three out of 38 participants (7.9%) dropped out during the walkway PBT and 2 out of 37 participants (5.4%) dropped out during the treadmill PBT (Figure 1).

The characteristics, falls, and usual gait parameters of the participants are summarised in Table 2. There were no differences between the W-T and T-W groups in the proportion of women or participant age, height, weight, body mass index, leg dominance, past falls, fear of falling, depressive symptoms, or gait parameters (*P* > 0.05).

**Table 2:** Participant characteristics, fall history, and usual gait parameters.

**Variables**	**Total sample**	**Group O-T**	**Group T-O**	***P***
	**(*n* = 38)**	**(*n* = 19)**	**(*n* = 19)**	
Age (years)	73.6 (4.7)	74.0 (5.1)	73.2 (4.3)	0.632
Sex, *N* (% female)	21 (55.3%)	11 (57.9%)	10 (52.6%)	0.744
Height (m)	1.69 (0.10)	1.69 (0.02)	1.69 (0.09)	0.992
Weight (kg)	74.3 (13.1)	75.2 (12.5)	73.4 (14.0)	0.674
BMI (kg/m^2^)	26.0 (3.5)	26.4 (3.4)	25.6 (3.6)	0.502
Dominant leg, *N* (% right)	38 (100%)	19 (100%)	19 (100%)	1.000
FES-I (score)	18.8 (3.6)	18.7 (4.4)	18.9 (2.8)	0.861
HAD (score)	3.95 (3.42)	3.68 (3.59)	4.21 (3.33)	0.642
Fallers, *N* (%)^*^	19 (50%)	11 (57.9%)	8 (42.1%)	0.330
Multiple fallers, *N* (%)^**^	10 (26.3%)	5 (26.3%)	5 (26.3%)	1.000
Step length (m)	0.65 (0.10)	0.65 (0.08)	0.64 (0.12)	0.877
Cadence (steps/min)	107.5 (9.0)	106.3 (7.4)	108.7 (10.4)	0.402
Gait speed (m/s)	1.16 (0.21)	1.15 (0.15)	1.17 (0.26)	0.709

*Data are mean (SD) or N (%). BMI, body mass index; FES-I, Falls Efficacy Scale-International; HAD, Hospital Anxiety and Depression scale*.

* *Number of people reporting at least 1 fall for the previous 12 months*.

** *Number of people reporting 2 or more falls for the previous 12 months*.

### Anxiety and Perceived Difficulty

On average, participants reported significantly higher anxiety during treadmill PBT compared to walkway PBT (1.82 ± 0.83 vs. 1.58 ± 0.59, *P* = 0.030). Average perceived difficulty scores during treadmill PBT were also significantly higher compared to walkway PBT (2.02 ± 0.74 vs. 1.65 ± 0.54, *P* = 0.001). There were no significant changes of anxiety or perceived difficulty over time during both training conditions, except for a significant decrease of perceived difficulty from N1 to N5 on the walkway (*P* = 0.04).

### Walkway PBT

**Figure 2:**
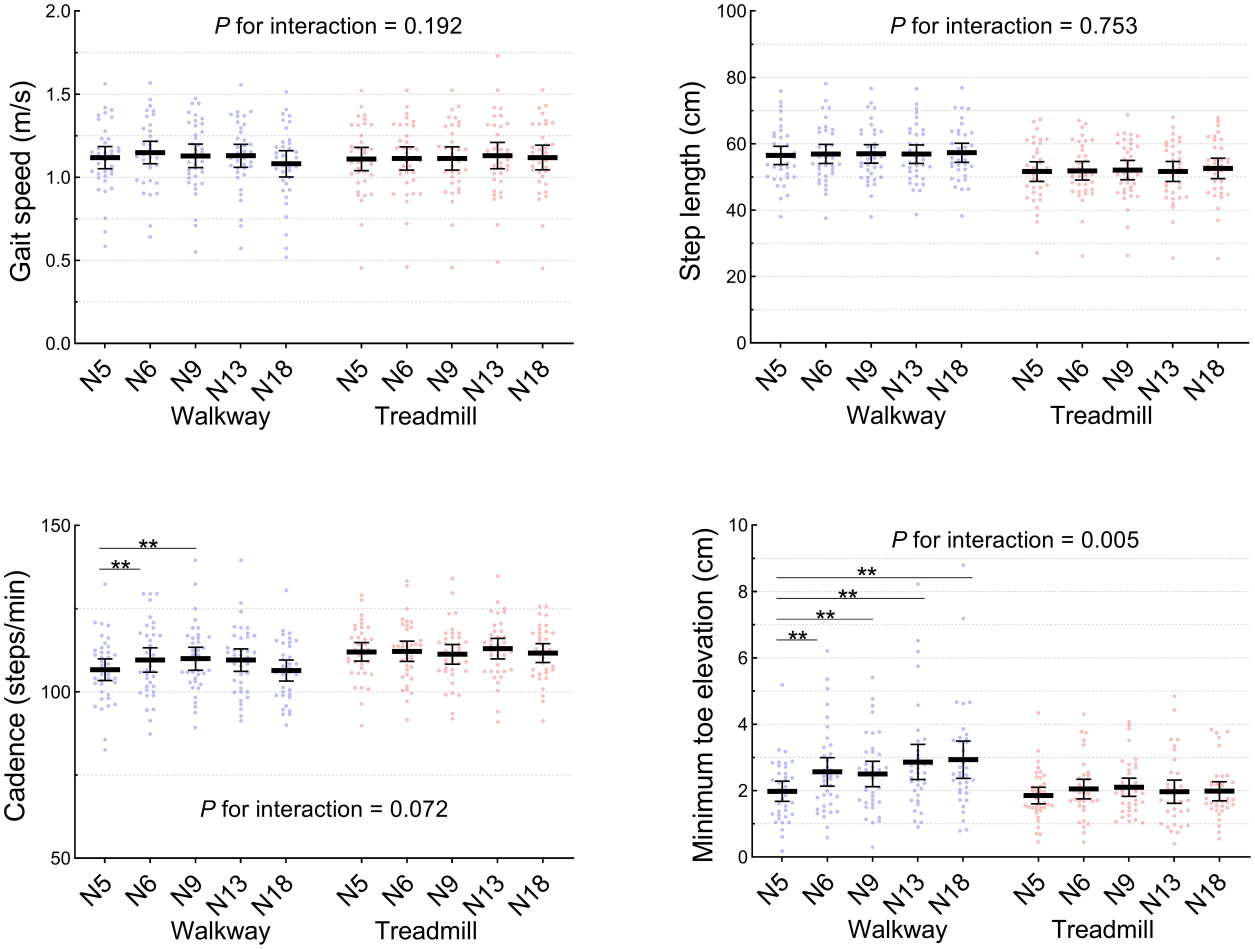
Spatiotemporal parameters during normal walks on the treadmill and overground walkway (*n* = 38). N5 and N6 were prior to and following the first trip, respectively. The middle and error bars represent mean and 95% confidence interval. **P < 0.01.

No significant differences in gait speed and step length were detected among the walkway normal walks (N5 vs. N6/N9/N13/N18, *P* > 0.05, Figure 2). Minimum toe elevation during normal walks in both groups was significantly increased in N6 (2.6 ± 1.3 cm), N9 (2.5 ± 1.1 cm), N13 (2.9 ± 1.6 cm), and N18 (2.9 ± 1.6 cm) compared to N5 (2.0 ± 0.9 cm) prior to the first trip (*P* < 0.01). A significant increase in walkway cadence was seen at N6 (110 ± 11.0 steps/min) and N9 (110 ± 10.3 steps/min) compared to N5 (107 ± 9.8 steps/min) (*P* < 0.05).

**Figure 3:**
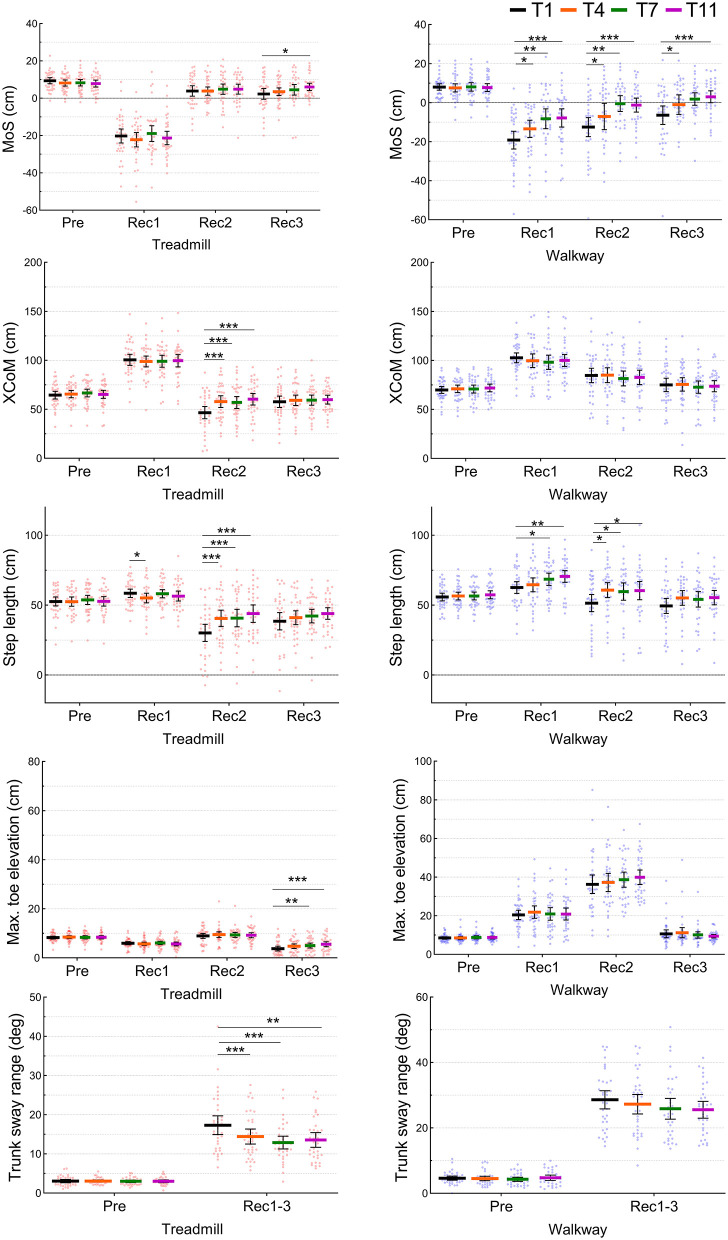
Kinematic parameters at one previous (Pre) and three recovery (Rec1, Rec2, and Rec3) steps during four trip trials (T1, T4, T7, and T11) on the treadmill and overground walkway (*n* = 38). The mid lines and error bars represent means and 95% confidence intervals. *P < 0.05, **P < 0.01, ***P < 0.001.

There were no changes in any of the kinematic parameters during the previous step in walkway trip trials (T1 vs. T4/T7/T11, *P* > 0.05) (Figure 3). A significant increase in step length was observed in Rec1 (T1: 62.9 ± 12.7 cm, T11: 70.5 ± 12.1 cm) and Rec2 (T1: 51.4 ± 18.6 cm, T11: 60.7 ± 15.9 cm) (*P* < 0.05). The MoS also significantly improved in Rec1 (T1: −19.2 ± 13.8 cm, T11: −7.8 ± 13.6 cm), Rec2 (T1: −12.5 ± 14.8 cm, T11: −1.2 ± 10.4 cm), and Rec3 (T1: −6.4 ± 14.4 cm, T11: 3.0 ± 8.9 cm) (*P* < 0.01). No significant changes over time were found in recovery step *XCoM*, trunk sway range, and maximum toe elevation on the walkway (*P* > 0.05).

### Treadmill PBT

During the treadmill normal walks, no significant changes were observed in any of the spatiotemporal gait parameters over time (N5 vs. N6/N9/N13/N18, *P* > 0.05) (Figure 2). Similarly, during the belt acceleration trials on the treadmill, there were no significant changes in the previous step kinematic parameters (*P* > 0.05) (Figure 3). A significant increase from T1 to T11 was found in Rec3 MoS (T1: 2.3 ± 8.7 cm, T11: 6.1 ± 5.6 cm), Rec2 step length (T1: 30.1 ± 18.3 cm, T11: 43.9 ± 18.1 cm), Rec2 *XCoM* (T1: 46.6 ± 18.7 cm, T11: 60.3 ± 17.4 cm), and Rec3 maximum toe elevation (T1: 3.8 ± 2.9 cm, T11: 5.6 ± 2.9 cm). A significant reduction in recovery trunk sway range on the treadmill was observed from T1 (17.3 ± 7.2 deg) and T11 (13.5 ± 5.4 deg) (*P* < 0.05).

### Interactions Between Time and Condition

A significant time- and condition-interaction was detected in MoS (*P* < 0.001) indicating greater improvements during the walkway PBT, compared to during treadmill PBT. Another significant interaction in *XCoM* (*P* = 0.024) indicated a greater increase in treadmill PBT than walkway PBT. No significant interactions were observed in step length, maximum toe elevation, and trunk sway range (*P* > 0.05).

### Transfer of Training Adaptations From the Treadmill PBT to a Walkway Trip

**Figure 4:**
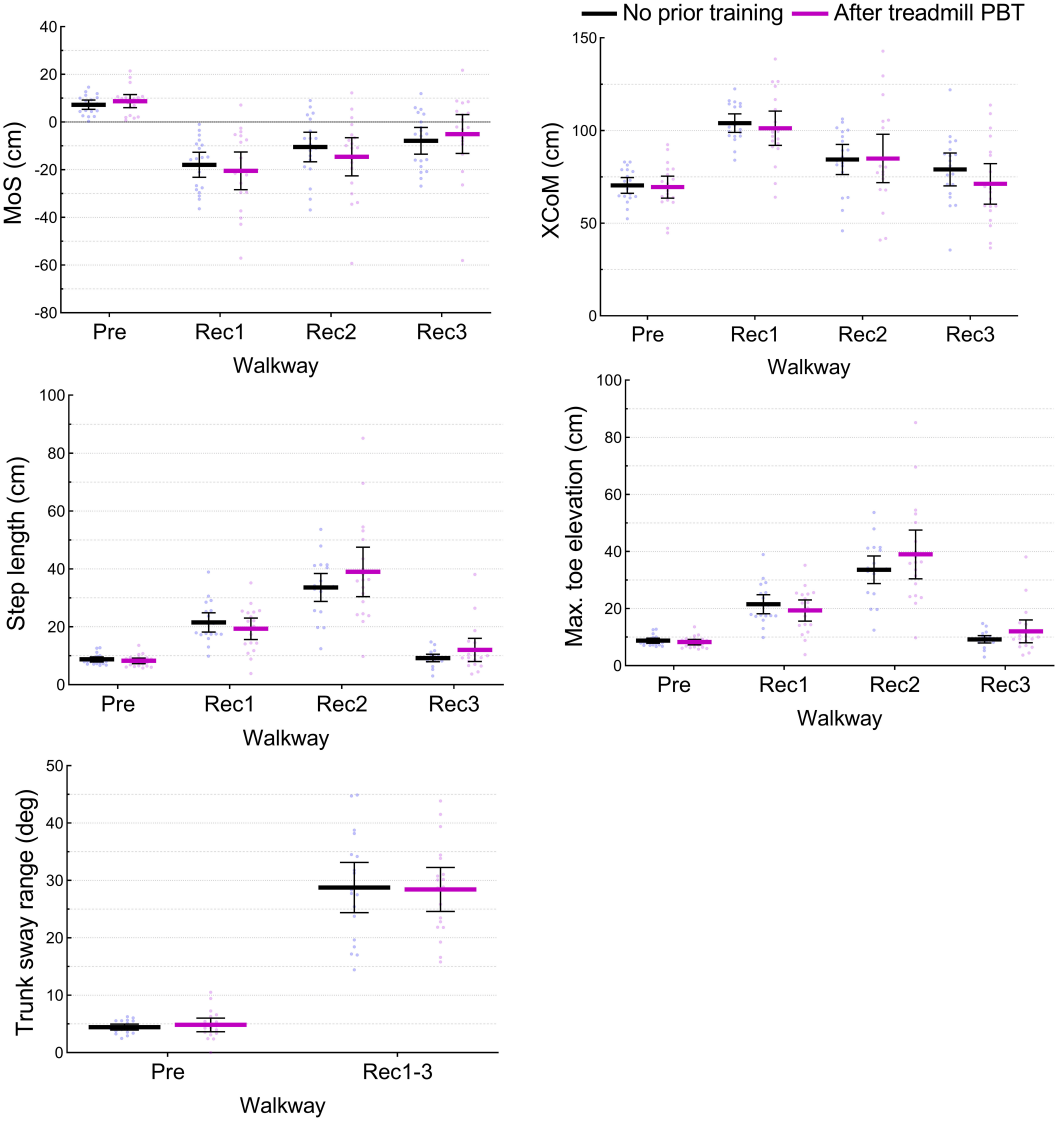
Test of transfer from treadmill PBT to an actual trip. Kinematic parameters at one previous (Pre) and three recovery (Rec1, Rec2, and Rec3) steps during the first walkway trip (T1) were compared between Group T-W (who had previously completed treadmill PBT) and Group W-T (who had no prior training). The mid lines and error bars represent means and 95% confidence intervals. No significant differences were detected (*P* > 0.05).

During the first walkway trip (T1), there were no significant differences in any kinematic parameters in any steps between Group T-W (who had previously completed treadmill PBT) and Group W-T (who had no prior training) (*P* > 0.05) (Figure 4).

## Discussion

This cross-over trial is the first to directly compare PBT involving walkway trips against PBT involving belt accelerations on a treadmill. The walkway PBT resulted in improved dynamic stability and greater step length following a trip, which supports our first hypothesis. The treadmill PBT also resulted in improved MoS, *XCoM*, step length, and less trunk sway following a belt acceleration, but contrary to our second hypothesis, treadmill PBT did not transfer to better recovery to a first trip on the walkway.

### Adaptations to PBT Using Trips on a Walkway

This trial demonstrated that older adults could improve their balance recovery following walkway trips. A similar increase in dynamic stability has been reported by previous studies that trained young and older adults with 8–24 walkway trips (Wang et al., 2012, 2020; Bhatt et al., 2013). However, previous studies administered all trips to the left foot in a fixed location resulting in a significant predictive gait adaptation seen as increased toe elevation (8–10 cm) (Wang et al., 2012, 2020; Bhatt et al., 2013) and the majority (12–60%) of participants avoided the obstacle on the last trip. In contrast, our walkway method maintained a high level of unpredictability in repeated trials by randomly inducing trips to both feet in various hidden locations. Therefore, we detected no predictive gait adaptations except for a small increase in minimum toe elevation (0.9 ± 1.1 cm) during normal walk trials. Maximum toe elevation in the previous step was 8.6 ± 2.1 cm and the tripping board was sufficiently high (14 cm) to induce legitimate trips to examine reactive adaptation during balance recovery. The unchanged gait speed, recovery step *XCoM*, and trunk sway suggest that the magnitude of balance perturbation induced by the trips was constant throughout the repeated trials. Thus, the increased MoS during the recovery step likely reflects the improved balance recovery response to trips. The walkway trips involved obstruction of the swing foot, substantial forward shift of *XCoM*, and trunk sway. Thus, it was necessary to take a longer recovery step (i.e., base of support) (Okubo et al., 2018) to provide a counter torque to catch the falling upper body. The ability to rapidly generate an extensor moment and position in the recovery limb has been identified as one of the key intrinsic limitations to balance recovery in older adults (van Dieen et al., 2005). This study shows that unexpected walkway trips can successfully train older adults to take longer recovery steps to increase the likelihood of balance recovery.

### Adaptations to PBT Using Treadmill Belt Accelerations

The improvement in MoS was found in the third recovery step over the 11 treadmill belt accelerations (8 m/s^2^ to 200% of walking speed). This is consistent with a previous study that exposed young and older adults to 10 belt accelerations (3 m/s^2^ to 180% of walking speed) and found improved MoS during the third to fifth recovery steps (McCrum et al., 2020). We also found increased *XCoM* and step length in the second recovery step which also replicated the results reported by McCrum et al. who also reported full retention over 1 month (McCrum et al., 2018). A reduction in trunk sway during recovery steps was also found as reactive balance adaptation to repeated exposure to treadmill belt accelerations. This agrees with a study in 16 stroke patients who underwent a single session of 15 treadmill perturbations from standing (22 cm displacement, acceleration/deceleration ±13.89 m/s^2^, velocity 0.56 m/s) and reported a reduction in trunk flexion but no improvement in MoS (Nevisipour et al., 2019). An RCT of 30 older adults who walked on a treadmill also reported an improvement in trunk control (i.e., reduction in trunk velocity) following both anterior–posterior (deceleration −9 m/s^2^ for 0.12 s) and medio-lateral (displacement 5 cm in 0.31 s) perturbations, which was retained after 1 week (Rieger et al., 2020). Interestingly, they found no difference between the intervention (16 perturbations) and control group indicating exposure to eight perturbations during the baseline assessment was sufficient to improve trunk control. This rapid adaptation coincides with our finding showing a reduction in trunk sway by T4 and T7. It is possible that the body has rapidly adapted to relax and reduce stiffness and thus less momentum is transferred from the foot on the suddenly accelerated treadmill belt to trunk flexion. Thus, these significant improvements in MoS, *XCoM*, recovery step length, and trunk sway reaffirm there is some capacity for reactive adaptation during treadmill PBT but the benefit of such adaptation needs to be examined.

### Transfer From Treadmill PBT to an Actual Trip

Following completion of treadmill PBT (Group T-W), the response to the first walkway trip was not significantly different from those with no prior training (Group W-T). Treadmill PBT has high clinical feasibility requiring less space, time, and human resources compared to walkway PBT. However, our findings indicate that the adaptation to treadmill belt accelerations may not improve recovery from real-life overground trips; likely because treadmill PBT did not provide the motor skills to deal with obstacles. A small increase in maximal toe elevation (on average 3.8 cm in T1 to 5.6 cm in T11 in Rec3) during the treadmill PBT was clearly not sufficient during the actual trip that required much higher foot elevation (on average 20.4 cm in Rec1, 26.3 cm in Rec2, 10.6 cm in Rec3 in T1). Our findings contrast to a previous study conducted on 34 young adults reporting significant beneficial effects of treadmill slip training on overground slip recovery (Yang et al., 2013). Such differences in transferability of slip and trip recovery training effects between treadmill and walkway PBT likely reflect the degree of shared biomechanical properties. Simulated slips induced by the deceleration or reverse rotation of the treadmill belt can replicate forward slipping of the leading foot. However, belt accelerations on the treadmill involve rapidly shifting the stance foot backward to induce forward rotation of the upper body, which requires rapid reactive stepping. Although the overall body response may be similar, a treadmill belt acceleration differs from an overground trip where the swing foot is physically obstructed requiring immediate elevation or lowering of the foot (Eng et al., 1994). It is likely that adaptations induced by PBT are highly task-specific and the greater the difference in biomechanical properties of the training, the more limited the transferability of training effects across different conditions. Indeed, König et al. found no transfer of training effects from treadmill trip training using ankle cable pulls to performance on a lean-and-release task that did not involve obstruction of the foot (Konig et al., 2019).

Two studies have reported training obstacle-clearing from an initial stance position on a treadmill can improve balance responses to actual trips. Grabiner et al. conducted an RCT involving 52 healthy middle-aged women in which intervention participants were trained to recover from sudden treadmill accelerations from rest (stance) by stepping over a 5 cm high foam obstacle (Grabiner et al., 2012). They found that following 120–150 treadmill-induced perturbations over 4 weeks, intervention participants had significantly fewer falls on an overground trip test compared to controls. Similar findings were reported by Bieryla et al. in that a training program involving 20 treadmill accelerations from rest requiring a step over a 7.6 cm high obstacle produced improved trunk control during an overground test trip in a small trial of 12 older adults (Bieryla et al., 2007). It is also possible to administer obstacle-induced trips on a treadmill by dropping an obstacle onto the belt (King et al., 2019) but the increased complexity limits its feasibility in clinical settings. An instrumented treadmill that provides belt accelerations may be a useful way to train balance responses to backward slips at heel strikes (Yang et al., 2013) and forward slips at the late stance phase (Debelle et al., 2020) but may not be sufficient in preparing older adults for an actual trip. Further refinement of treadmill PBT protocols including belt kinematics and/or methods of delivering foot obstruction, as well as determination of optimal training doses and longer-term follow-ups are required to better clarify the clinical role of treadmill PBT training.

### Anxiety and Perceived Difficulty

Anxiety can negatively affect reactive balance control (e.g., delayed and more rigid responses) (Carpenter et al., 2004; Okubo et al., 2021), and thus should be minimised for better training outcomes. However, only a few studies have quantified anxiety during PBT (Okubo et al., 2019a,b). Anxiety and perceived difficulty were higher during PBT on a treadmill compared to PBT on the walkway. Since the magnitude of perturbations induced by the treadmill was not greater than that on the walkway, this higher anxiety and perceived difficulty were likely due to unfamiliarity to treadmill walking and the elevated surface of the large, instrumented treadmill. The provision of a surrounding platform at the level of the treadmill belt surface may assist in reducing anxiety during treadmill PBT.

### Limitations

This study has some limitations that warrant attention. First, study participants were healthy older adults who may not be representative of the older population. Older adults in poorer health or with increased fear of falling may show lower acceptability to PBT. Second, whilst we used the walkway trip as a surrogate for real-world trips, our study findings should be verified with a sufficient sample size and follow-up for evaluating the effect of PBT on falls in daily life.

## Conclusions

This study demonstrated that older adults can learn to improve dynamic stability and stepping by repeated exposure to walkway trips. Exposure to belt accelerations on the treadmill may also improve dynamic stability, stepping, and trunk control in older adults. However, these adaptations obtained on a treadmill are likely not generalisable to an overground trip. Further refinement of treadmill trip training protocols to improve ecological authenticity while maintaining clinical feasibility is required.

## Data Availability Statement

The datasets analysed for this study can be provided to upon reasonable request approved by the University of New South Wales Human Research Ethics Committee. Requests to access the datasets should be directed to yoshiro_okubo@yahoo.co.jp.

## Ethics Statement

The studies involving human participants were reviewed and approved by University of New South Wales Human Research Ethics Committee. The participants provided their written informed consent to participate in this study.

## Author Contributions

YO, DS, PS, and SL contributed to the conception of the study. YO, PS, and DS contributed to the experimental setup. PS, YO, and MD conducted the participant recruitment, data acquisition, and processing. PS and YO conducted statistical analyses and drafted the manuscript. DS, MD, and SL revised the article for important intellectual content. All authors approved the final version.

## Conflict of Interest

The authors declare that the research was conducted in the absence of any commercial or financial relationships that could be construed as a potential conflict of interest.

## Publisher's Note

All claims expressed in this article are solely those of the authors and do not necessarily represent those of their affiliated organizations, or those of the publisher, the editors and the reviewers. Any product that may be evaluated in this article, or claim that may be made by its manufacturer, is not guaranteed or endorsed by the publisher.
