# Supplementary material for: Perturbation-Based Balance Training Using Repeated Trips on a Walkway vs. Belt Accelerations on a Treadmill: A Cross-Over Randomised Controlled Trial in Community-Dwelling Older Adults
Source: Front Sports Act Living. 2021 Aug 20;3:702320. doi: 10.3389/fspor.2021.702320 (PMC8417700; doi:10.3389/fspor.2021.702320)
Supplement: Supplementary file 1 [file Table_1.pdf]

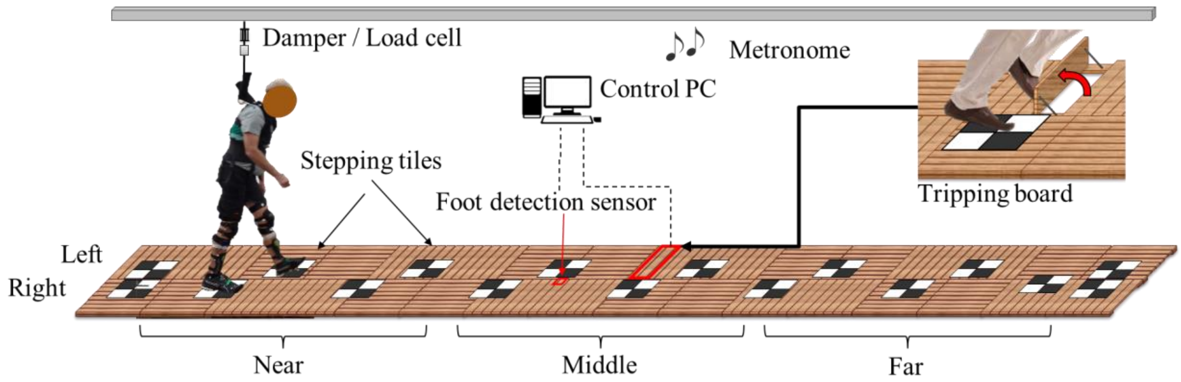

Supplementary Figure 1. The trip walkway used for perturbation-based balance training. Black and white target stepping tiles were placed according to individual's usual step length and a metronome was set to the individual's usual cadence. A trip was induced using a 14cm height tripping board that was activated by the optical foot detection sensor catching the foot in the late swing phase. The trip location was varied to be in the near, middle or far zone.

Supplementary Table 1 Definitions of biomechanical parameters related to the treadmill and walkway.

Parameter	Condition	Definition
Normal walks		
Step length (cm)	Walkway and Treadmill	Average of anteroposterior distance between left and right ankle markers (lateral malleolus) at foot-strike (Supakkul 2017).
Cadence (steps/min)	Walkway and Treadmill	Derived from the number of steps taken during a trial divided by time, transformed to steps per minute.
Gait velocity (m/s)	Walkway	Calculated from the difference in anteroposterior position of the centre of mass between the first and last foot strike over time, transformed to m/s.
Gait velocity (m/s)	Treadmill	Calculated using anteroposterior velocity of left and right heel markers according to Souza et al. (2017).
Minimum toe elevation	Walkway and Treadmill	Vertical difference between swing and stance toe marker when the toe marker is minimum height during the swing.
Maximum toe elevation	Walkway and Treadmill	Vertical difference between swing and stance toe marker when swing toe marker is maximum height during first swing phase of the trial.
Events in trip trials		
Previous step	Walkway	The last uninterrupted step which ends with the last foot strike before trip onset.

Previous step	Treadmill	The last uninterrupted step which ends with the foot strike at perturbation onset.
Trip onset	Walkway	The moment in time when the foot first contacts the tripping obstacle during swing phase.
Perturbation onset	Treadmill	The moment in time when the foot strikes the treadmill belt that has accelerated.
Recovery step	Walkway and Treadmill<br><br>Trip with **lowering** strategy	The step that ends with the 2nd foot strike after trip/perturbation onset (= 1st foot strike).
Recovery step	Walkway<br><br>Trip with **elevating** strategy	The step which ends with the 1st foot strike after trip/perturbation onset.
<u>Kinematics</u>		
Stepping strategy	Walkway	Determined by the anteroposterior position of the ankle marker of the perturbed leg relative to the obstacle at time of first foot strike after trip onset. If the ankle marker remains posterior to the obstacle, then the trial is classified as lowering.<br><br>If the ankle marker is anterior to the obstacle, then the trial is classified as elevating.
Stepping strategy	Treadmill	The method of treadmill perturbation does not allow an elevating strategy thus all treadmill perturbed trials are assigned lowering strategy.
Extrapolated Centre of Mass position ($XCoM_{position}$)	Walkway	Calculated using anteroposterior position of centre of mass (CoMposition), anteroposterior velocity of centre of mass (VCoM: calculated from difference in anteroposterior position of centre of mass between consecutive frames and the framerate), gravitational acceleration (g: 9.81 m/s2) and leg length (L: calculated as distance between centre of mass and leading ankle marker in the sagittal plane) according to (Süptitz et al. 2013)
Extrapolated Centre of Mass position (XCoMposition)	Treadmill	Calculated using anteroposterior position of centre of mass, anteroposterior velocity of centre of mass (calculated from difference in anteroposterior position of centre of mass between consecutive frames and the framerate), velocity of base of support and leg length (calculated as distance between centre of mass and leading ankle marker in the sagittal plane) according to (Süptitz et al. 2013)

Velocity of Base of Support	Treadmill	Calculated by averaging instantaneous anteroposterior velocities of a heel marker during the stance phase (between two foot strikes). The velocities were calculated from difference in anteroposterior position of heel markers between consecutive frames and the framerate) during the stance phase.
Extrapolated Centre of Mass (XCoM)	Walkway and Treadmill	Calculated as the difference in anteroposterior position of the $XCoM_{position}$ and ankle marker of rear stance leg. A positive and negative value denotes that the extrapolated centre of mass is positioned anterior and posterior to the rear foot ankle, respectively.
Anterior border of Base of Support	Walkway and Treadmill	Defined as the leading edge of the leading foot in the sagittal plane, taken as the toe marker plus 30% of the anteroposterior distance between the toe and heel markers.
Margin of Stability (MoS)	Walkway and Treadmill	Calculated as difference in anteroposterior position of extrapolated centre of mass position and anterior border of base of support.<br><br>If the extrapolated centre of mass position is distal to Anterior border of Base of Support, then the Margin of Stability will have a negative value to denote an unstable state.<br><br>If the Extrapolated Centre of Mass position is posterior to Anterior border of Base of Support, then the Margin of Stability will have a positive value to denote a stable state.
Trunk sway range	Walkway and Treadmill	Calculated as absolute difference between maximum and minimum trunk angles in the sagittal plane during the Previous step, or during the Recovery step and next two steps.

Souza, GSDSE, Rodrigues, FB, Andrade, AO & Vieira, MF 2017, 'A simple, reliable method to determine the mean gait speed using heel markers on a treadmill', Computer Methods in Biomechanics and Biomedical Engineering, vol. 20, no. 8, pp. 901-904, DOI:10.1080/10255842.2017.1309395.

Supakkul, K 2017, 'Using positional heel-marker data to more accurately calculate stride length for treadmill walking: a step length approach', arXiv preprint arXiv:1710.09030.

Süptitz, F, Catalá, MM, Brüggemann, G-P & Karamanidis, K 2013, 'Dynamic stability control during perturbed walking can be assessed by a reduced kinematic model across the adult female lifespan', Human Movement Science, vol. 32, no. 6, pp. 1404-1414, DOI:https://doi.org/10.1016/j.humov.2013.07.008.

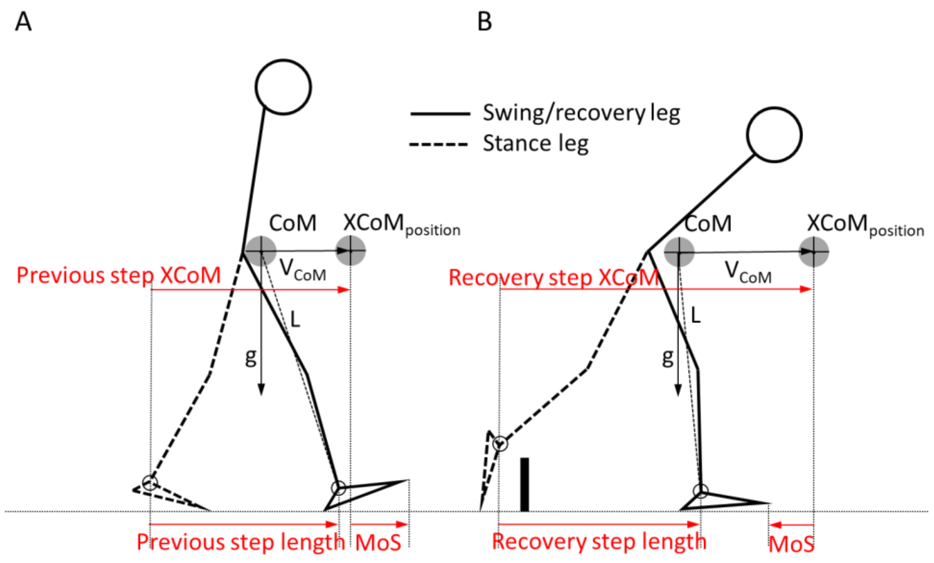

Supplementary Figure 2: Schematic illustration of the inverted pendulum model applied to the previous step (A) and recovery step (B) in trip trials.

The inverted pendulum model consists of the centre of mass (CoM) and pendulum length (L). The CoM has a velocity V_{CoM} which when added to the current CoM position yields the extrapolated centre of mass position ($XCoM_{position}$). The reported extrapolated centre of mass (XCoM) was calculated as the anteroposterior difference between the $XCoM_{position}$ and trailing ankle marker. The margin of stability (MoS) was calculated as difference between $XCoM_{position}$ and the closest edge of base of support which was usually the anterior edge of the leading foot. The step length was taken as the anteroposterior difference in ankle marker positions at foot strike.
